# Eating Behaviours of British University Students: A Cluster Analysis on a Neglected Issue

**DOI:** 10.1155/2015/639239

**Published:** 2015-10-13

**Authors:** Jina Tanton, Lorna J. Dodd, Lorayne Woodfield, Mzwandile Mabhala

**Affiliations:** ^1^Department of Physical Education and Sport Studies, Newman University, Genners Lane, Bartley Green, Birmingham B32 3NT, UK; ^2^Department of Psychology & Counselling, Newman University, Genners Lane, Bartley Green, Birmingham B32 3NT, UK; ^3^Faculty of Health and Social Care, University of Chester, Castle Drive, Chester CH1 1SL, UK

## Abstract

Unhealthy diet is a primary risk factor for noncommunicable diseases. University student populations are known to engage in health risking lifestyle behaviours including risky eating behaviours. The purpose of this study was to examine eating behaviour patterns in a population of British university students using a two-step cluster analysis. Consumption prevalence of snack, convenience, and fast foods in addition to fruit and vegetables was measured using a self-report “Student Eating Behaviours” questionnaire on 345 undergraduate university students. Four clusters were identified: “risky eating behaviours,” “mixed eating behaviours,” “moderate eating behaviours,” and “favourable eating behaviours.” Nineteen percent of students were categorised as having “favourable eating behaviours” whilst just under a third of students were categorised within the two most risky clusters. Riskier eating behaviour patterns were associated with living on campus and Christian faith. The findings of this study highlight the importance of university microenvironments on eating behaviours in university student populations. Religion as a mediator of eating behaviours is a novel finding.

## 1. Introduction

Noncommunicable diseases (NCDs) continue to be the leading cause of chronic illness, disability, and mortality globally [1]. An unhealthy diet is one of the four preventable primary risk factors for NCDs [2]. Low fibre intake and excessive fat intake are reported as distal risk factors for overweight and obesity, which in turn are intermediate risk factors for NCDs [3]. Fast foods and convenience foods are often low in nutritional value although energy dense [4]. Furthermore, higher consumption of convenience and fast foods has been associated with a lower intake of fruit and vegetables [5, 6] and lower diet quality [7]. Sufficient consumption of fruit and vegetables is important as the nutritional content of fruit and vegetables, such as dietary fibre, vitamins, and minerals, is associated with a reduced risk of cardiovascular disease and type II diabetes [8]. University student populations are widely reported to engage in unhealthy lifestyle behaviours including unhealthy eating behaviours such as high consumption of snack foods [9–13], consumption of convenience foods [7], high consumption of fast foods [5, 7, 11, 13–16], and insufficient consumption of fruit and vegetables [9, 11, 12, 14–26]. Thus, students indulging in these behaviours may be at increased risk of weight gain and future development of NCDs.

Comparison of studies examining the prevalence of eating behaviours in student populations is difficult due to the different ways in which eating behaviours have been measured and reported and differences in the demographic characteristics of the students sampled. That said, trends are beginning to emerge that suggest cause for concern. Published figures suggest more than a third of students consume snack foods “at least several times a week” [11, 12] or 3-4 times a week or more [13].

The reported prevalence of fast food consumption, three or more times per week [5, 14], “at least several times per week” [11], and 3-4 times a week or more [13], is varied, ranging from 20.2% in Polish university students [13] to 46% in USA university students [5]. Of interest, using the criteria of two of more takeaway meals as a main meal per week, Thorpe et al. [7] reported only 12.5% of Australian university students to meet the criteria. The lower prevalence despite a more acute criterion may be explained by the specification that takeaway meals must have been consumed as a main meal to be included in the data [7] or may reflect cultural differences between Australian university students and students of other countries, such as what has been demonstrated by El Ansari et al. [11].

World Health Organisation (WHO) and United Kingdom (UK) guidelines recommend a minimum consumption of five portions of fruit and vegetables each day. Average daily consumption by university students has been found to range from 2.2 to 3.8 portions per day [14, 17, 19, 20, 25, 26]. The prevalence of students meeting current fruit and vegetable consumption guidelines is low ranging from 3.27% to 34.7% [12, 18, 21–24].

Only one study [7] to the authors' knowledge has reported on the consumption of convenience food in a student population. Examining the behaviours of an Australian university student population, Thorpe et al. [7] reported 30% of students to consume a convenience meal as a main meal at least once per week [7].

Eating behaviours have been reported to differ by sex [9, 12] and living arrangement [11, 27] in university student populations. Moreno-Gómez et al. [9] reported diet quality to be higher in females, whilst El Ansari et al. [12] found recommended consumption of fruit and vegetables and consumption of sweet items such as chocolate and candy to be higher amongst female students. El Ansari et al. [11] and Papadaki et al. [27] found students living away from the parental home to have poorer eating habits for most indicators.

Despite evidence demonstrating that health and lifestyle behaviours coexist [28–35], few studies have examined the clustering of health and lifestyle behaviours in university student populations [16, 19, 36]. Only one study to the authors' knowledge included more than one indicator of eating behaviour [16]. Consequently, no study to date has examined solely the clustering of eating behaviours in a university student population. Cluster analysis technique enables subgroups with shared characteristics to be identified within a population [19]. Examining how such behaviours cluster together and the impact of demographic and university microenvironment factors on eating behaviours is important, particularly as the presence of multiple unhealthy lifestyle behaviours contributes to multiplicative rather than additive health risk [12].

Presently, resources to address the growing prevalence of NCDs are stretched [3]. Thus, in order to reduce future prevalence of NCDs, preventative action is required [37]. University students are of interest as they present a large, captive population of emerging adults [38, 39] who are expected to fulfil influential roles in society as teachers, policy makers, and professionals [19]. The years spent in university education have been promoted as a time for supporting emerging adults to develop health promoting lifestyle behaviours [16]. The transition into university education is significant as during this period emerging adults experience greater freedom to make choices regarding their health and lifestyle behaviours [17, 40, 41]. Furthermore, many students find themselves in a new environment [18, 41] and experience changes to support networks and social norms [18, 42, 43]. Consequently, transition in living environment is likely to alter eating behaviours [11, 44]. As decision makers and role models, the attitudes and behaviours adopted by graduates during their university education have the potential to have further reaching impact on wider society [45] and therefore the health and lifestyle behaviours of university students are of public health interest [19, 45].

The limited research on students' unhealthy eating behaviours is not conclusive. Clarity of eating behaviour patterns is essential in this population to ensure that appropriate interventions are introduced which will encourage health promoting eating behaviour practices [46]. Research on this area needs to go beyond just reporting the unhealthy and healthy eating behaviours students undertake but move towards demonstrating how eating behaviours relate to each other and how student characteristics and environment can impact upon such practices [46]. Despite prevalence of risky eating behaviours in student populations, there is a lack of research examining the clustering of health risking and health promoting eating behaviours using cluster analysis technique in both UK and international university student populations. Minimal research has examined the dietary behaviours of European university students [46]. Therefore the aims of this study were twofold: to examine the eating behaviour patterns of a university student population using cluster analysis and to identify demographic and university microenvironment correlates of student eating behaviour patterns.

## 2. Method

### 2.1. Sample and Procedure

Data collection took place in a single English university with an undergraduate population of 1,707 undergraduate students. Three hundred and forty-five undergraduate students (20.2% of the population) volunteered to complete a “Student Eating Behaviour Questionnaire.” Questionnaires were administered during lecture time. Data was collected across the academic year of 2014-2015. Ethical approval was received from the institutional research ethics committee prior to data collection. Students were provided with an information sheet and consent form to complete before completing the self-report questionnaire. Sample characteristics are presented in Table 1.

### 2.2. Measures

A self-administered survey titled “Student Eating Behaviours” was developed based on previously validated questions that had been used within the literature. The questionnaire included questions on the following.

*Demographic Characteristics*. Demographic information was collected on age, sex, ethnicity, self-reported height and weight (from which BMI was calculated), religion, living arrangement, and year of study.

*Eating Behaviours*. Students were asked, “During the* last seven* days how many times* per day* have you eaten the following foods?” [11]. Students were required to indicate the number of portions of fruit, vegetables, and snack foods (e.g., chocolate, sweets, crisps, and cakes) they had consumed. Students were also asked, “During the last* seven days* how many times* per week* have you eaten the following foods?” Students were asked to indicate the number of convenience meals (e.g., microwave meals and oven ready foods such as pizza and chicken nuggets) and fast food or takeaway meals (e.g., Chinese, Indian, and Thai takeaway food, fish and chips, fried chicken, and McDonald's) they had consumed. Reported numbers of fruit and vegetables were combined to allow comparison against current UK guidelines.

*Statistical Analysis*. A two-step cluster analysis was used to identify clusters based on four eating behaviours. Two-step cluster analysis was chosen as it is appropriate for both continuous and categorical data and data sets larger than 200 [47]. Analyses including chi-square, to identify differences between the clusters with regard to demographic characteristics, and MANOVA, to identify differences between each of the eating behaviours within the clusters, were employed. A Bonferroni adjusted *p* value was used for the MANOVA to correct for multiple comparisons. All analyses were conducted using the Statistical Package for Social Sciences (Version 22, SPSS Inc., Chicago, IL, USA).

## 3. Results

### 3.1. Descriptive Statistics

Three hundred and forty-five British undergraduate students (66% female; 71% white; 49% Christian; 82% living off campus; 44% first year of study) volunteered to complete a questionnaire. BMI was defined by the American College of Sports Medicine (2010) criteria. Mean BMI was 23.5 ± 4.0. Sixty-nine percent of students were classified as normal weight by BMI; 25.9% were classified as overweight or obese. Demographic characteristics are shown in Table 1.

### 3.2. Cluster Analysis

The cluster analysis technique revealed four distinct clusters (Table 2). Cluster 1 (risky eating behaviours) was characterised by high snacking, high consumption of convenience and fast foods, and low consumption of fruit and vegetables. Cluster 2 (mixed eating behaviours) was characterised by high snacking, high consumption of convenience and fast foods, and moderate consumption of fruit and vegetables. Cluster 3 (moderate eating behaviours) was characterised by low snacking, moderate consumption of convenience and fast foods, and low consumption of fruit and vegetables. Cluster 4 (favourable eating behaviours) was characterised by moderate snacking, low consumption of convenience and fast foods, and high consumption of fruit and vegetables. Cluster 4 was the only group to meet current UK recommendations [48, 49] for fruit and vegetable intake.

Significant differences were found between the clusters, across religion (*χ* ^2^_(2)_ = 32.824, *p* < 0.01, and Cramer's phi = .313) and living arrangement (*χ* ^2^_(2)_ = 13.140, *p* < 0.01, and Cramer's phi = .196), but no significant differences were observed for age, sex, BMI, ethnicity, or year of study (*p* > 0.05) (see Table 2). These findings should be considered in accordance with the sample size. Significant associations between clusters 1 (risky eating behaviours) and 2 (mixed eating behaviours), clusters 1 and 3 (moderate eating behaviours), clusters 1 and 4 (favourable eating behaviours), and clusters 2 and 3 and religion were observed with a higher percentage of Christian students found in cluster 1 and cluster 3. However, no significant associations were found between clusters 2 and 4 or clusters 3 and 4 and religion. Data are presented in Table 3.

Significant differences between clusters 1 and 2 and clusters 1 and 4 and living arrangement were observed with a higher percentage of students living on campus found in cluster 1. Significant associations between clusters 2 and 3 and living arrangement were observed with cluster 3 being characterised by both a higher percentage of students living on campus and a higher percentage of students living off campus. Significant associations between clusters 3 and 4 and living arrangement were observed with a higher percentage of students living on campus found in cluster 3. No significant associations were found between clusters 1 and 3 or clusters 2 and 4 and living arrangement. Data are presented in Table 3.

MANOVA revealed significant differences between the clusters and eating behaviours (*F*_(8,810)_ = 103.910, *p* < 0.0125, Pillai's trace = 1.650, and partial eta squared = .550—large effect). Follow-up post hoc tests revealed significant differences (*p* < 0.0125) between the clusters (see Table 2).

## 4. Discussion

Unhealthy diet is one of the four primary preventable risk factors for NCDs [50]. Furthermore, unhealthy diet is a known risk factor for overweight and obesity [3]. Findings of this study demonstrate distinct cluster patterns of eating behaviours in a British university student population. Based on the eating behaviours measured, four distinct clusters were identified: cluster 1: risky eating behaviours, cluster 2: mixed eating behaviours, cluster 3: moderate eating behaviours, and cluster 4: favourable eating behaviours. Only 18.6% of the sample was grouped within the favourable eating cluster. Just under a third of the sample (31.6%) fell within cluster 1 (risky eating behaviour) or cluster 2 (mixed eating behaviour); the two clusters are characterised by the most risky eating behaviours. The high prevalence of unhealthy eating behaviour patterns demonstrates the need for interventions promoting healthy eating behaviour patterns amongst British university students [46].

Snack and convenience and fast food consumption were clearly shown to cluster together with a high prevalence of these behaviours characterising cluster 1 and a low prevalence of these behaviours characterising cluster 4, with significant differences observed for fruit and vegetable and convenience and fast food consumption. Furthermore, clear distinctions between cluster 2 (mixed eating behaviours) and cluster 3 (moderate eating behaviours) can be observed with significant differences for snack and convenience food consumption.

In contrast to previous research on diet and eating behaviours, clusters were found not to differ by sex although differences were observed by living arrangement and religion. A higher proportion of students living on campus were found in cluster 1 (risky eating behaviours) and cluster 3 (moderate eating behaviours). Research has reported students living outside of the family home to consume fewer fruit and vegetables [11, 27]. In agreement with this, both clusters 1 and 3 were characterised by low fruit and vegetable consumption. Eighty-two percent of students reporting to live off campus also reported living with a parent or guardian. In terms of snack and fast food consumption El Ansari et al. [11] reported living arrangement not to influence consumption; however the findings of this study are less clear. Whilst cluster 1 (risky eating behaviours) is characterised by a higher consumption of snack and fast foods and a higher percentage of students living on campus, cluster 3 (moderate eating behaviours) is characterised by a lower consumption of snack and fast foods and is characterised by both a high percentage of students living on campus and a high percentage of students living off campus. Thus, the relationship between snack and fast food consumption is not clear and further investigation is required.

Religion has been reported to have a protective effect against health and lifestyle risk behaviours including risky alcohol consumption [51–53] and drug use [54, 55]; however religion has not previously been shown to be associated with healthy and unhealthy eating behaviours. The current study found cluster 1 (risky eating behaviours) and cluster 3 (moderate eating behaviours) to be categorized by a higher percentage of Christian students. Of interest, findings of Berry et al. [56] found students of Christian faith to report levels of binge drinking and sexual activity exceeding those of the wider student population sampled, including students of Muslim and Jewish faiths, leading to the suggestion that Christianity may offer less protection against riskful health and lifestyle behaviours than other religious faiths. A possible explanation for this may be the cultural expectations of specific religious groups [57]. Religion may support healthy lifestyle choices through mechanisms such as culture [58], social support, and prescription of expected behaviours [59].

The findings of this study reaffirm the role of the university microenvironment, particularly on campus living, in eating behaviours in university student populations. Suggested explanations for this include financial restrictions [11], availability of healthy meals [11], and food availability on campus [46, 60]. Whilst further research is needed to understand students' eating behaviour choices, current understanding would support a review of university food environments in sight of the recognised importance of supporting and developing health promoting eating behaviours in emerging adult populations.

The findings of this study should be considered with acknowledgement of the limitations. In comparison to other studies examining health behaviours in university student populations, the findings of this study are based on a relatively small sample size. Sample size was influenced by the total number of undergraduates at the chosen university and is sufficient for the analyses chosen. Thus the relatively small sample should be taken into consideration when reviewing the findings, especially within the analyses that assessed differences between the clusters and the separate factors, for example, gender and BMI. Data was collected by means of a self-report questionnaire and therefore recall error is possible. Furthermore, behaviours during the last seven days may not be representative of typical behaviour. Data reported is cross-sectional and therefore causation cannot be inferred. Finally clusters identified are population specific and thus the findings cannot be generalised [19].

Unhealthy and healthy eating behaviours have been shown to cluster together in an English university student population. Moreover riskier patterns of eating behaviour were observed in students living on campus and of Christian faith. Universities have a duty of care to their students and therefore the finding that students who spend greater amounts of time on campus are engaging in riskier eating behaviours should be cause for concern for university leaders. Further understanding of the factors shaping the eating behaviours of students living on English university campuses including analysis of university microenvironments is needed. Research to affirm the relationship and to clarify the mechanisms (e.g., social support and cultural expectation) underpinning the relationship between religion and lifestyle behaviours may enable lessons to be learnt that can foster health promoting behaviours.

## Figures and Tables

**Table 1:** Sample characteristics.

Age (years) (mean (SD))	21.4 (4.7)
Sex (*n* (%))	
Male	117 (33.9)
Female	228 (66.1)
BMI (kg·m^−2^) (mean ± SD)	23.5 (4.0)
BMI classification (%)	
Underweight	5.0
Normal weight	69.1
Overweight	19.6
Obese	6.3
Ethnicity (%)	
White	70.9
Mixed	5.2
Asian or Asian British	17.2
Black British	4.1
Chinese	0.3
Other	2.3
Religion (%)	
Christian	48.8
Hindu	1.5
Muslim	15.2
Sikh	2.1
Atheist	26.2
Other	6.3
Living arrangement (%)	
On campus	18.1
Off campus	81.9
Year of study (%)	
1	43.8
2	28.1
3	26.4
4	1.4
5	0.3

**Table 2:** Mean scores and percentages for the four clusters of British students at a UK university in 2014-2015.

	Cluster 1	Cluster 2	Cluster 3	Cluster 4
	(*n* = 64/18.6%)	(*n* = 45/13.0%)	(*n* = 172/49.9%)	(*n* = 64/18.6%)
	Risky eating behaviours	Mixed eating behaviours	Moderate eating behaviours	Favourable eating behaviours
	Mean (%)			
*Eating behaviours*				
Snacking (per day)	2.03^(b)^	4.69^(a,d,e)^	1.29	1.59
Convenience food consumption (per week)	7.07^(a,b,c)^	2.53^(d,e)^	1.47	0.96
Fast food consumption (per week)	4.2^(a,b,c)^	1.89^(d,e)^	1.19	0.85
Fruit and vegetable consumption (per day)	2.88	3.44	2.69	7.10^(c,e,f)^
*Demographic factors*				
Religion				*χ* ^2^_(2)_ = 32.824, phi = .313^*∗∗*^
Christian	26.2	12.2	45.7	15.9
Hindu	0.0	0.0	80.0	20.0
Muslim	19.6	23.5	39.2	17.6
Sikh	0	42.9	28.6	28.6
Atheist	8.0	10.2	61.4	20.5
Other	19.0	0.0	57.1	23.8
Living arrangement				*χ* ^2^_(2)_ = 13.140, phi = .196^*∗∗*^
On campus	27.1	4.8	59.7	8.1
Off campus	16.4	15.0	47.9	20.7
Age				*χ* ^2^_(2)_ = 11.455, phi = .182
Sex				*χ* ^2^_(2)_ = 6.905, phi = .141
BMI				*χ* ^2^_(2)_ = 12.992, phi = .208
Ethnicity				*χ* ^2^_(2)_ = 17.235, phi = .224

^*∗*^ *p* < 0.05, ^*∗∗*^ *p* < 0.01, MANOVA: (a) denotes significantly higher consumption when comparing clusters 1 and 2, (b) denotes significantly higher consumption when comparing clusters 1 and 3, (c) denotes significantly higher consumption when comparing clusters 1 and 4, (d) denotes significantly higher consumption when comparing clusters 2 and 3, (e) denotes significantly higher consumption when comparing clusters 2 and 4, and (f) denotes significantly higher consumption when comparing clusters 3 and 4.

**Table 3:** Between cluster differences.

	Cluster 1 versus 2		Cluster 1 versus 3		Cluster 1 versus 4	
	1	2	1	3	1	4
Religion	(*χ* ^2^_(2)_ = 12.556, *p* < 0.05, and Cramer's phi = .341)		(*χ* ^2^_(2)_ = 15.350, *p* < 0.01, and Cramer's phi = .258)		(*χ* ^2^_(2)_ = 12.127, *p* < 0.05, and Cramer's phi = .311)	
Christian (%)	68.3	31.7	36.4	63.6	62.3	37.7
Hindu (%)	0.0	0.0	0.0	100.0	0.0	100.0
Muslim (%)	45.5	54.5	33.3	66.7	52.6	47.4
Sikh (%)	0.0	100.0	0.0	100.0	0.0	100.0
Atheist (%)	43.8	56.3	11.5	88.5	28.0	72.0
Other (%)	100.0	0.0	25.0	75.0	44.4	55.6

Living arrangement	(*χ* ^2^_(2)_ = 7.181, *p* < 0.01, and Cramer's phi = .258)		(*χ* ^2^_(2)_ = .741, *p* > 0.05, and Cramer's phi = .056)		(*χ* ^2^_(2)_ = 7.930, *p* < 0.01, and Cramer's phi = .251)	
On campus (%)	85.0	15.0	31.5	68.5	77.3	22.7
Off campus (%)	52.3	47.7	25.6	74.4	44.2	55.8

	Cluster 2 versus 3		Cluster 2 versus 4		Cluster 3 versus 4	
	2	3	2	4	3	4

Religion	(*χ* ^2^_(2)_ = 15.880, *p* < 0.01, and Cramer's phi = .274)		(*χ* ^2^_(2)_ = 7.865, *p* > 0.05, and Cramer's phi = .274)		(*χ* ^2^_(2)_ = 1.718, *p* > 0.05, and Cramer's phi = .087)	
Christian (%)	21.1	78.9	43.5	56.5	74.3	25.7
Hindu (%)	0.0	100.0	0.0	100.0	80.0	20.0
Muslim (%)	37.5	62.5	57.1	42.9	69.0	31.0
Sikh (%)	60.0	40.0	60.0	40.0	50.0	50.0
Atheist (%)	14.3	85.7	33.3	66.7	75.0	25.0
Other (%)	0.0	100.0	0.0	100.0	70.6	29.4

Living arrangement	(*χ* ^2^_(2)_ = 5.291, *p* < 0.05, and Cramer's phi = −.157)		(*χ* ^2^_(2)_ = 0.62, *p* > 0.05, and Cramer's phi = −.024)		(*χ* ^2^_(2)_ = 5.868, *p* < 0.05, and Cramer's phi = .158)	
On campus (%)	7.5	92.5	37.5	62.5	88.1	11.9
Off campus (%)	23.9	76.1	42.0	58.0	69.8	30.2

## References

[1] WHO (2013). *Global Action Plan for the Prevention and Control of Noncommunicable Diseases 2013–2020*.

[2] WHO (2008). *2008–2013 Action Plan for the Global Strategy for the Prevention and Control of Noncommunicable Diseases*.

[3] Cecchini M., Sassi F., Lauer J. A., Lee Y. Y., Guajardo-Barron V., Chisholm D. (2010). Tackling of unhealthy diets, physical inactivity, and obesity: health effects and cost-effectiveness. *The Lancet*.

[4] Racette S. B., Deusinger S. S., Deusinger R. H. (2003). Obesity: overview of prevalence, etiology, and treatment. *Physical Therapy*.

[5] Racette S. B., Deusinger S. S., Strube M. J., Highstein G. R., Deusinger R. H. (2005). Weight changes, exercise, and dietary patterns during freshman and sophomore years of college. *Journal of American College Health*.

[6] Paeratakul S., Ferdinand D. P., Champagne C. M., Ryan D. H., Bray G. A. (2003). Fast-food consumption among US adults and children: dietary and nutrient intake profile. *Journal of the American Dietetic Association*.

[7] Thorpe M. G., Kestin M., Riddell L. J., Keast R. S., McNaughton S. A. (2014). Diet quality in young adults and its association with food-related behaviours. *Public Health Nutrition*.

[8] Bazzano L. A. (2005). *Dietary Intake of Fruit and Vegetables and Risk of Diabetes Mellitus and Cardiovascular Diseases*.

[9] Moreno-Gómez C., Romaguera-Bosch D., Tauler-Riera P. (2012). Clustering of lifestyle factors in Spanish university students: the relationship between smoking, alcohol consumption, physical activity and diet quality. *Public Health Nutrition*.

[10] Wansink B., Cao Y., Saini P., Shimizu M., Just D. R. (2013). College cafeteria snack food purchases become less healthy with each passing week of the semester. *Public Health Nutrition*.

[11] El Ansari W., Stock C., Mikolajczyk R. T. (2012). Relationships between food consumption and living arrangements among university students in four European countries—a cross-sectional study. *Nutrition Journal*.

[12] El Ansari W., Stock C., John J. (2011). Health promoting behaviours and lifestyle characteristics of students at seven universities in the UK. *Central European Journal of Public Health*.

[13] Likus W., Milka D., Bajor G., Jachacz-Łopata M., Dorzak B. (2013). Dietary habits and physical activity in students from the Medical University of Silesia in Poland. *Roczniki Państwowego Zakładu Higieny*.

[14] Cooke R., Papadaki A. (2014). Nutrition label use mediates the positive relationship between nutrition knowledge and attitudes towards healthy eating with dietary quality among university students in the UK. *Appetite*.

[15] Ibrahim N. K., Mahnashi M., Al-Dhaheri A. (2014). Risk factors of coronary heart disease among medical students in King Abdulaziz University, Jeddah, Saudi Arabia. *BMC Public Health*.

[16] Al-Nakeeb Y., Lyons M., Dodd L., Al-Nuaim A. (2015). An investigation into the lifestyle, health habits and risk factors of young adults. *International Journal of Environmental Research and Public Health*.

[17] Small M., Bailey-Davis L., Morgan N., Maggs J. (2013). Changes in eating and physical activity behaviors across seven semesters of college: living on or off campus matters. *Health Education & Behavior*.

[18] Keller S., Maddock J. E., Hannöver W., Thyrian J. R., Basler H.-D. (2008). Multiple health risk behaviors in German first year university students. *Preventive Medicine*.

[19] Dodd L. J., Al-Nakeeb Y., Nevill A., Forshaw M. J. (2010). Lifestyle risk factors of students: a cluster analytical approach. *Preventive Medicine*.

[20] Tomasone J. R., Meikle N., Bray S. R. (2014). Intentions and trait self-control predict fruit and vegetable consumption during the transition to first-year university. *Journal of American College Health*.

[21] Alsunni A. A., Badar A. (2015). Fruit and vegetable consumption and its determinants among Saudi university students. *Journal of Taibah University Medical Sciences*.

[22] Al-Otaibi H. H. (2014). The pattern of fruit and vegetable consumption among Saudi university students. *Global Journal of Health Science*.

[23] Peltzer K., Pengpid S. (2015). Correlates of healthy fruit and vegetable diet in students in low, middle and high income countries. *International Journal of Public Health*.

[24] Elsoadaa S. S., Abdelhafez A. M., Rabeh N. M., Zahran S. E., Osfor M. M. H. (2013). Consumption of fruits and vegetables among Umm Al- Qura University students in Makkah, Saudi Arabia: a cross -section study. *Life Science Journal*.

[25] Shive S. E., Morris M. N. (2006). Evaluation of the energize your life! Social marketing campaign pilot study to increase fruit intake among community college students. *Journal of American College Health*.

[26] Evans R., Kawabata M., Thomas S. (2015). Prediction of fruit and vegetable intake: the importance of contextualizing motivation. *British Journal of Health Psychology*.

[27] Papadaki A., Hondros G., A. Scott J., Kapsokefalou M. (2007). Eating habits of University students living at, or away from home in Greece. *Appetite*.

[28] Burke V., Milligan R. A. K., Beilin L. J. (1997). Clustering of health-related behaviors among 18-year-old Australians. *Preventive Medicine*.

[29] Schuit A. J., Van Loon A. J. M., Tijhuis M., Ocké M. C. (2002). Clustering of lifestyle risk factors in a general adult population. *Preventive Medicine*.

[30] Pronk N. P., Anderson L. H., Crain A. L. (2004). Meeting recommendations for multiple healthy lifestyle factors: prevalence, clustering, and predictors among adolescent, adult, and senior health plan members. *American Journal of Preventive Medicine*.

[31] Chiolero A., Wietlisbach V., Ruffieux C., Paccaud F., Cornuz J. (2006). Clustering of risk behaviors with cigarette consumption: a population-based survey. *Preventive Medicine*.

[32] Poortinga W. (2007). The prevalence and clustering of four major lifestyle risk factors in an English adult population. *Preventive Medicine*.

[33] Sanchez A., Norman G. J., Sallis J. F., Calfas K. J., Cella J., Patrick K. (2007). Patterns and correlates of physical activity and nutrition behaviors in adolescents. *American Journal of Preventive Medicine*.

[34] Chou K.-L. (2008). The prevalence and clustering of four major lifestyle risk factors in Hong Kong Chinese older adults. *Journal of Aging and Health*.

[35] De Vries H., Kremers S., Smeets T., Reubsaet A. (2008). Clustering of diet, physical activity and smoking and a general willingness to change. *Psychology & Health*.

[36] Quintiliani L., Allen J., Marino M., Kelly-Weeder S., Li Y. (2010). Multiple health behavior clusters among female college students. *Patient Education and Counseling*.

[37] World Health Organization (2014). *Global Status Report on Noncommunicable Diseases 2014*.

[38] Burke J. D., Reilly R. A., Morrell J. S., Lofgren I. E. (2009). The university of New Hampshire's young adult health risk screening initiative. *Journal of the American Dietetic Association*.

[39] Morrell J. S., Lofgren I. E., Burke J. D., Reilly R. A. (2012). Metabolic syndrome, obesity, and related risk factors among college men and women. *Journal of American College Health*.

[40] Lee R. L. T., Loke A. J. T. Y. (2005). Health-promoting behaviors and psychosocial well-being of university students in Hong Kong. *Public Health Nursing*.

[41] Steyl T., Phillips J. (2011). Actual and perceived substance use of health science students at a university in the Western Cape, South Africa. *African Health Sciences*.

[42] Bray S. R., Kwan M. Y. W. (2006). Physical activity is associated with better health and psychological well-being during transition to university life. *Journal of American College Health*.

[43] Sebena R., El Ansari W., Stock C., Orosova O., Mikolajczyk R. T. (2012). Are perceived stress, depressive symptoms and religiosity associated with alcohol consumption? A survey of freshmen university students across five European countries. *Substance Abuse: Treatment, Prevention, and Policy*.

[44] Beasley L. J., Hackett A. F., Maxwell S. M. (2004). The dietary and health behaviour of young people aged 18-25 years living independently or in the family home in Liverpool, UK. *International Journal of Consumer Studies*.

[45] El Ansari W., Stock C. (2012). Factors associated with smoking, quit attempts and attitudes towards total smoking bans at university: a survey of seven universities in England, Wales and Northern Ireland. *Asian Pacific Journal of Cancer Prevention*.

[46] Deliens T., Clarys P., De Bourdeaudhuij I., Deforche B. (2014). Determinants of eating behaviour in university students: a qualitative study using focus group discussions. *BMC Public Health*.

[47] Bittmann R. M., Gelbard R. M. (2007). Decision-making method using a visual approach for cluster analysis problems; indicative classification algorithms and grouping scope. *Expert Systems*.

[48] World Health Organization (2015). *Healthy Diet*.

[49] Department of Health (2010). *5 A Day Introduction*.

[50] WHO (2008). 2008–2013 Action plan for the global strategy for the prevention and control of noncommunicable diseases: the six objectives of the 2008–2013 action plan are. *Blood*.

[51] El Ansari W., Sebena R., Stock C. (2014). Do importance of religious faith and healthy lifestyle modify the relationships between depressive symptoms and four indicators of alcohol consumption? A survey of students across seven universities in England, Wales, and Northern Ireland. *Substance Use and Misuse*.

[52] Neighbors C., Brown G. A., Dibello A. M., Rodriguez L. M., Foster D. W. (2013). Reliance on God, prayer, and religion reduces influence of perceived norms on drinking. *Journal of Studies on Alcohol and Drugs*.

[53] Atwell K., Abraham C., Duka T. (2011). A parsimonious, integrative model of key psychological correlates of UK university students' alcohol consumption. *Alcohol and Alcoholism*.

[54] Gomes F. C., de Andrade A. G., Izbicki R., Almeida A. M., de Oliveira L. G. (2013). Religion as a protective factor against drug use among Brazilian university students: a national survey. *Revista Brasileira de Psiquiatria*.

[55] Suerken C. K., Reboussin B. A., Sutfin E. L., Wagoner K. G., Spangler J., Wolfson M. (2014). Prevalence of marijuana use at college entry and risk factors for initiation during freshman year. *Addictive Behaviors*.

[56] Berry D., Bass C. P., Shimp-Fassler C., Succop P. (2013). Risk, religiosity, and emerging adulthood: description of Christian, Jewish, and Muslim university students at entering the freshman year. *Mental Health, Religion and Culture*.

[57] Shatenstein B., Ghadirian P. (1998). Influence on diet, health behaviors and their outcome in select ethnocultural and religious groups. *Nutrition*.

[58] Eckersley R. M. (2007). Culture, spirituality, religion and health: looking at the big picture. *The Medical Journal of Australia*.

[59] Williams D. R., Sternthal M. J. (2007). Spirituality, religion and health: evidence and research directions. *Medical Journal of Australia*.

[60] Symonds C. R., Martins A. C., Hartwell H. J. (2013). Foodscapes and wellbeing in the workplace: a university setting. *Nutrition & Food Science*.

